# Risk factors of anastomosis‐related difficult endoscopic retrograde cholangiopancreatography following endoscopic ultrasound‐guided gastro‐gastrostomy using a standardized protocol (with video)

**DOI:** 10.1111/den.14544

**Published:** 2023-04-23

**Authors:** Enrique Pérez‐Cuadrado‐Robles, Hadrien Alric, Lucille Quénéhervé, Laurent Monino, Tigran Poghosyan, Hedi Benosman, Ariane Vienne, Guillaume Perrod, Lionel Rebibo, Ali Aidibi, Elena Tenorio‐González, Emilia Ragot, Mehdi Karoui, Christophe Cellier, Gabriel Rahmi

**Affiliations:** ^1^ >Department of Gastroenterology Georges‐Pompidou European Hospital, Assistance Publique‐Hôpitaux de Paris Paris France; ^2^ Department of Surgery Georges‐Pompidou European Hospital, Assistance Publique‐Hôpitaux de Paris Paris France; ^3^ Paris Cité University Paris France; ^4^ Department of Surgery Bichat Hospital, Assistance Publique‐Hôpitaux de Paris Paris France; ^5^ Department of Gastroenterology University Hospital of Brest Brest France; ^6^ Department of Gastroenterology and Hepatology Université catholique de Louvain, Cliniques universitaires Saint‐Luc Brussels Belgium

**Keywords:** anastomosis, bypass, EDGE, endoscopic ultrasound, LAMS

## Abstract

**Objectives:**

Little is known about how to perform the endoscopic ultrasound (EUS)‐directed transgastric endoscopic retrograde cholangiopancreatography (ERCP; EDGE) in patients with gastric bypass using lumen‐apposing metal stents (LAMS). The aim was to assess the risk factors of anastomosis‐related difficult ERCP.

**Methods:**

Observational single‐center study. All patients who underwent an EDGE procedure in 2020–2022 following a standardized protocol were included. Risk factors for difficult ERCP, defined as the need of >5 min LAMS dilation or failure to pass a duodenoscope in the second duodenum, were assessed.

**Results:**

Forty‐five ERCPs were performed in 31 patients (57.4 ± 8.2 years old, 38.7% male). The EUS procedure was done using a wire‐guided technique (*n* = 28, 90.3%) for biliary stones (*n* = 22, 71%) in most cases. The location of the anastomosis was gastro‐gastric (*n* = 24, 77.4%) and mainly in the middle‐excluded stomach (*n* = 21, 67.7%) with an oblique axis (*n* = 22, 71%). The ERCP technical success was 96.8%. There were 10 difficult ERCPs (32.3%) due to timing (*n* = 8), anastomotic dilation (*n* = 8), or failure to pass (*n* = 3). By multivariable analysis adjusted by two‐stage procedures, the risk factors for a difficult ERCP were the jejuno‐gastric route (85.7% vs. 16.7%; odds ratio [OR_a_] 31.875; 95% confidence interval [CI] 1.649–616.155; *P* = 0.022), and the anastomosis to the proximal/distal excluded stomach (70% vs. 14.3%; OR_a_ 22.667; 95% CI 1.676–306.570; *P* = 0.019). There was only one complication (3.2%) and one persistent gastro‐gastric fistula (3.2%) in a median follow‐up of 4 months (2–18 months), with no weight regain (*P* = 0.465).

**Conclusions:**

The jejunogastric route and the anastomosis with the proximal/distal excluded stomach during the EDGE procedure increase the difficulty of ERCP.

## INTRODUCTION

Endoscopic ultrasound (EUS)‐directed transgastric interventions (EDGIs) are becoming the new paradigm in the treatment of patients with gastric bypass,^1^, ^2^ achieving high rates of technical success and low rates of severe adverse events (AEs).^3^, ^4^ The EUS‐directed transgastric endoscopic retrograde cholangiopancreatography procedure (ERCP; EDGE) using lumen‐apposing metal stents (LAMS) is gaining ground in this setting^5^, ^6^ and has been proposed as an alternative in this setting by the European Society of Gastrointestinal Endoscopy (ESGE), overcoming the invasiveness of laparoscopy‐assisted ERCP (LA‐ERCP) and the limitations of enteroscopy‐assisted ERCP (EA‐ERCP).^2^ LA‐ERCP provides a safe alternative with less pain compared to open surgery,^7^ with fewer complications but longer procedure times.^8^ In addition, the surgical approach has been described as more effective than EA‐ERCP, but associated with a higher rate of AEs.^9^ Similarly, a recent systematic review and meta‐analysis concluded that LA‐ERCP and EDGE are associated with higher technical and therapeutic success compared to EA‐ERCP, though accompanied with more AEs.^10^ Most procedure‐related AEs are minor,^11^ and the most common is stent migration. LAMS dwell time has been described as a significant predictor of persistent fistula, increasing the risk by 9.5% for every week that the stent is left in situ.^12^ In addition, the use of smaller diameter LAMS (15 mm) could be a predictor of stent migration.^13^ Conversely, a lower AE rate using large‐bore stents has been reported.^14^

Although the EUS‐guided gastro‐gastrostomy is an overall safe technique and the EDGE procedure has high clinical success rates, the passage of the duodenoscope through the endoscopic anastomosis and further positioning in the excluded duodenum can be challenging and affect the procedure time, the risk of AEs, and the technical success.^15^ Little is known about how and where the anastomosis should be performed and the ideal location and stent axis to prevent stent migration or to facilitate the duodenoscope passage. Furthermore, different studies evaluated this procedure considering different techniques (e.g., wire‐guided, freehand LAMS insertion), timings (e.g., ERCP 48 h–15 days following EUS‐guided anastomosis) and stent sizes (15–20 mm), therefore the interpretation of their results in daily practice can be difficult. Finally, the real technical/clinical success rates of the EDGE are probably overestimated in the literature as most published studies are retrospective series with a small number of procedures in highly experienced centers.

Thus, the difficulty of the ERCP procedure in these cases could not be related to a difficult biliary cannulation and the classical associated factors,^16^ but to the patient's anatomy and the characteristics of the recently created endoscopic anastomosis. Indeed, the concept of gastro‐gastric‐related “difficult ERCP” during the EDGE intervention has never been evaluated to the best of our knowledge. The aim of the present study was to assess the risk factors of difficult ERCP following EDGE using a standardized protocol.

## METHODS

### Patients

This is an observational retrospective single‐center study using a prospectively collected database. All consecutive adult patients with a gastric bypass who underwent an EDGE at Georges‐Pompidou European Hospital under the ANMOD network (“Interventional endoscopy on modified anatomy”) between January 2020 and December 2022 were included. Those patients who underwent a gastro‐gastrostomy for other reasons other than subsequent ERCP were excluded. The protocol was submitted to the Local Ethical Committee (CERUPHO).

Age and sex variables were collected. Baseline characteristics such as the primary indication, the presence of acute cholangitis, the length of hospital stay, and the presence of ascites were noted. The weight before the EDGE procedure and at the end of the follow‐up was collected as well as the time from the bypass surgery to the EDGE procedure.

### EDGE procedure

**Figure 1:**
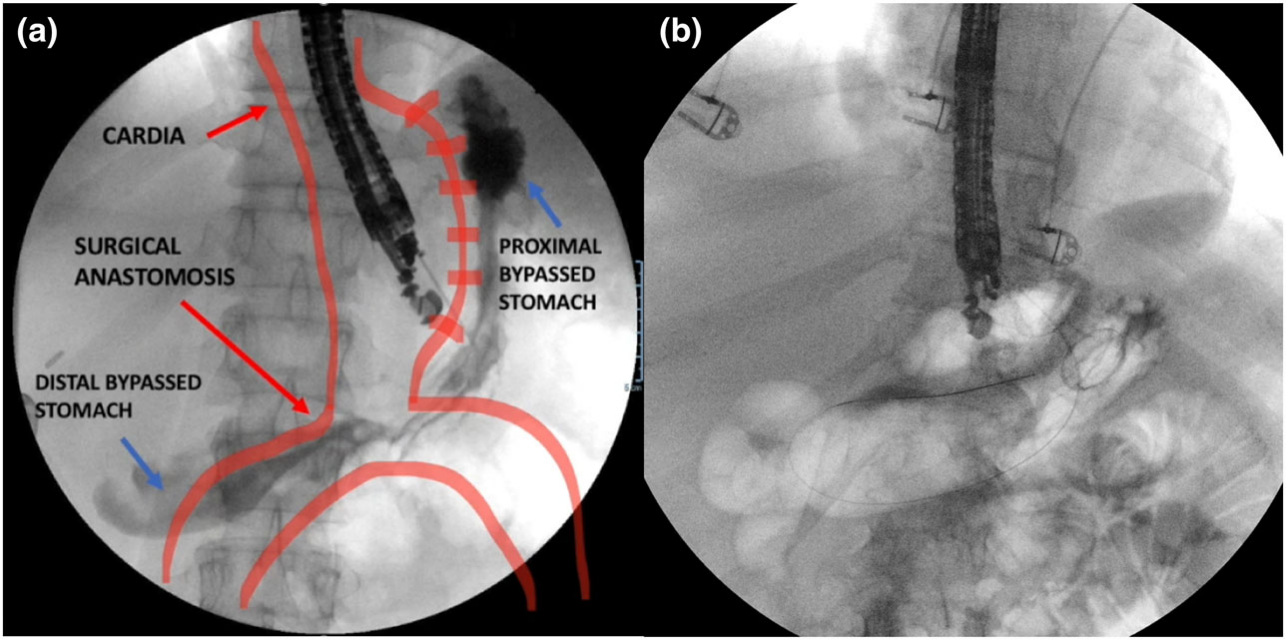
(a) First endoscopic ultrasound (EUS) puncture of the middle stomach with opacification to understand the postsurgical anatomy in a patient with a gastric bypass to decide the better location of the subsequent EUS‐guided gastro‐gastric anastomosis. (b) Of note, the proximal and distal bypassed stomach are more dilated compared to the middle gastric region, but this location was preferred to have a gastro‐gastric oblique axis, ensuring an easy passage for further transgastric interventions.

First, a linear‐array echoendoscope (GF‐UCT180; Olympus, Tokyo, Japan) was used to create the anastomosis. A standardized protocol was used as follows: the surgical gastrojejunal anastomosis was identified to avoid performing the endoscopic anastomosis through the jejunal limb. The echoendoscope was positioned above the surgical anastomosis and at least 2 cm below the cardia if possible. A 19G needle was used to perform a puncture of the excluded stomach and an opacification was done to understand the anatomy of the patient under fluoroscopy control without trying to fill in or dilate the gastric cavity. The optimal location for the anastomosis was chosen in the greater curvature of the excluded stomach avoiding the proximal bypassed stomach or the pre‐pyloric antrum if possible (Fig. 1). A second puncture was done if the first was not in an ideal position based on the opacification. Thus, a 0.025/0.035 inch guidewire was passed and a wire‐guided gastro‐gastric perforation with an electrocautery enhanced LAMS (20 × 10 mm HotAxios; Boston Scientific, Voisins‐le‐Bretonneux, France) was performed. No stent dilation was performed in the two‐stage procedures. Procedure time was collected.

Second, an ERCP procedure was performed at 48–72 h. The axis of the stent was evaluated horizontal, oblique, vertical. The duodenoscope was gently passed through the LAMS using torquing movements under fluoroscopy.

**Figure 2:**
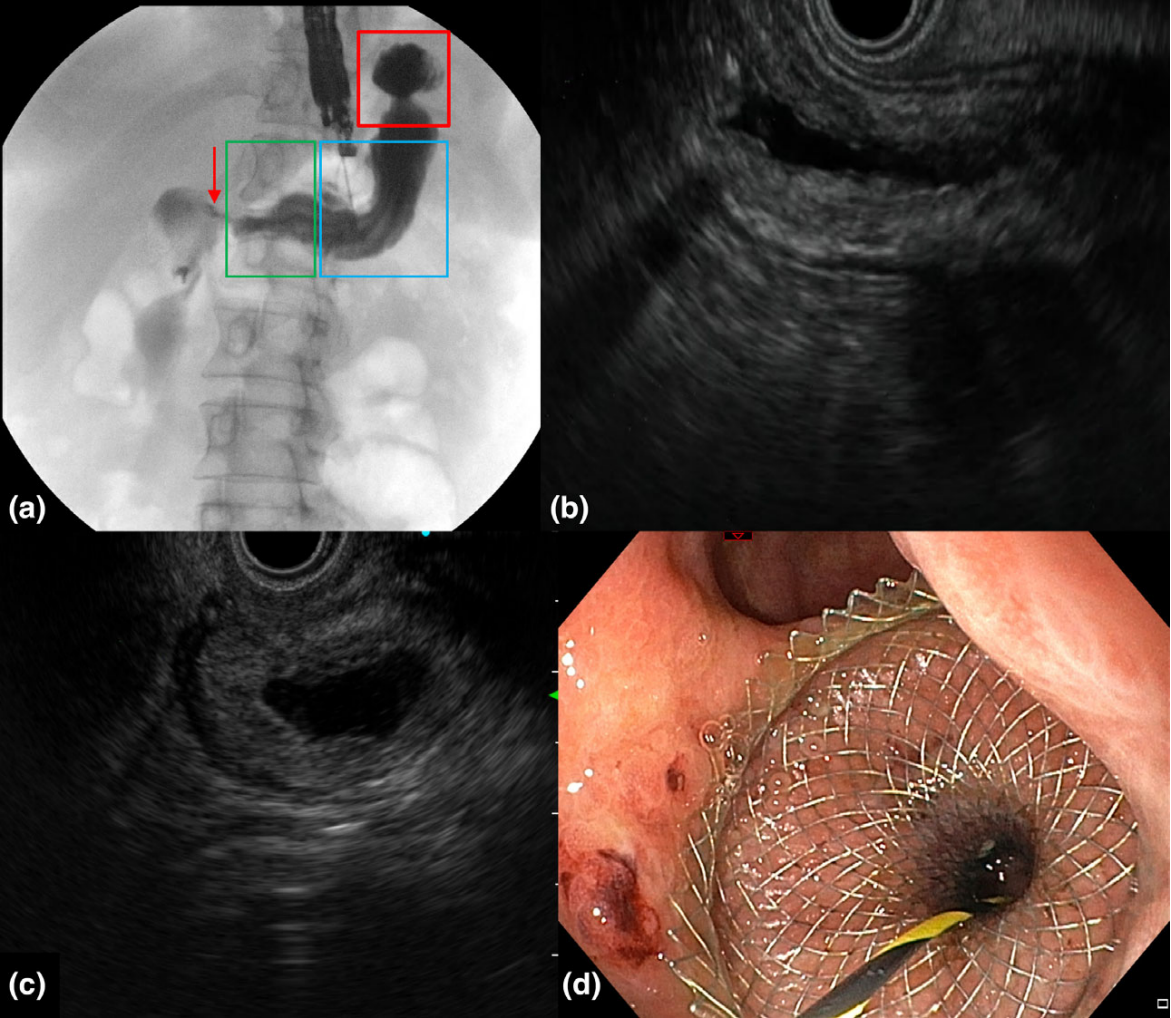
(a) Endoscopic ultrasound transgastric puncture with opacification under fluoroscopy guidance of the excluded stomach in a patient with a gastric bypass prior to deciding the better location and axis of the gastro‐gastric anastomosis. The proximal excluded stomach (red square), middle‐excluded stomach (blue square), distal‐excluded stomach (green square), and pylorus (arrow) can be identified. (b) Endoscopic ultrasound image of the middle‐excluded stomach, usually less dilated and with a larger horizontal size compared to the (c) proximal‐excluded gastric cavity. (d) Endoscopic image of a lumen‐apposing metal stent deployed in transgastric position a few centimeters above the surgical gastro‐jejunal anastomosis.

All procedures were performed under general anesthesia and CO_2_ insufflation. The location of the endoscopic anastomosis (gastro‐gastric vs. jejuno‐gastric; proximal‐, middle‐ or distal‐excluded stomach) and the LAMS axis were prospectively assessed by consensus of two authors (EPCR, HA) based on the videos/pictures (Fig. 2). The number of EUS punctures was also considered.

Adverse events were evaluated as described in the ESGE guidelines^2^ and graded using the AGREE classification.^17^ The LAMS dislodgement during the procedure was also noted. All EUS‐guided procedures were performed by a single endoscopist (EPCR).

### Outcomes and definitions

The primary outcome was to analyze the risk factors of a difficult passage to the excluded duodenum (difficult ERCP). Only the first ERCP procedure per patient was considered. A difficult passage to the duodenum was defined as a composite outcome including:

1. A time >5 min to achieve a short position of the duodenoscope in the second duodenum.
2. Dilation of the LAMS to pass the anastomosis (two‐stage procedures).
3. The failure to pass the gastro‐gastric anastomosis with a duodenoscope and/or further use of a forward‐viewing endoscope for performing ERCP.

The secondary outcomes were the technical and clinical success of the ERCP. Clinical success was defined by the normalization of bilirubin and symptomatic relief 1 week after the procedure.

### Follow‐up

An endoscopic and clinical follow‐up was considered. LAMS removal was planned 3–4 weeks following the EDGE. The gastro‐gastric fistula was not systematically closed. The jejuno‐gastric fistulas were closed using over‐the‐scope clips due to the theoretically higher risk of persistent fistula and argon plasma coagulation was performed if bleeding was identified following stent removal. A follow‐up upper gastrointestinal (GI) endoscopy was performed at 2–4 months to confirm the absence of persistent fistula.

### Statistical analysis

Categorical variables were compared using the χ^2^‐test. Non‐normally distributed continuous variables were analyzed by Mann–Whitney *U*‐test and McNemar test. Normal and non‐normal variables were presented as mean (SD) and median (range). A multivariable analysis by binary logistic regression was carried out to determine the risk factors of difficult ERCP. All statistically significant variables in the univariable analysis were included in the model. A two‐sided *P*‐value < 0.05 was considered statistically significant. SPSS software version 24 was used (IBM, Armonk, NY, USA).

## RESULTS

### Patients

Thirty‐three patients underwent an EUS‐guided gastro‐gastrostomy. Two patients were excluded because of EUS (*n* = 1) or upper GI endoscopy (*n* = 1) interventions (EDGIs) without further ERCP. Finally, 45 ERCPs were performed in 31 patients (57.4 ± 8.2 years old, 38.7% male) at a median of 1 ERCP (range 1–3). The most common indication was biliary stones (*n* = 22, 71%), followed by biliary obstruction related to cholangiocarcinoma (*n* = 2, 6.5%) or ampullary lesion (*n* = 2, 6.5%), biliary leak (*n* = 2, 6.5%), sphincter of Oddi dysfunction (*n* = 1, 3.2%), and biliary stricture (*n* = 1, 3.2%). About half of patients (*n* = 14, 45.2%) presented with acute cholangitis at symptom onset, but only one of them was hospitalized in the intensive care unit. The median time from the bypass surgery to the EDGE procedure was 6 years (range 1–13). There was ascites in only one case (3.2%).

### Procedure

The EUS‐guided anastomosis was performed using a wire‐guided technique (*n* = 28, 90.3%) with a gastro‐gastric approach (*n* = 24, 77.4%) in most cases (Table 1). The jejuno‐gastric location was chosen when the excluded stomach was far away (>1 cm) from the transductor in transgastric position (*n* = 4) or to avoid a gastro‐gastric “horizontal” anastomosis with the proximal bypassed stomach in the subcardial region for these patients with a small gastric pouch (*n* = 3). The stent was mainly located in the middle‐excluded stomach (*n* = 21, 67.7%) with an oblique axis (*n* = 22, 71%). These features were confirmed by EUS and fluoroscopy images. There were six patients who underwent more than one EUS‐guided puncture to choose a better location of the anastomosis (19.4%; Video S1). The ERCP was performed with success in all but one case (96.8%), at a median of 3 days (range 2–19) following the EUS‐guided gastro‐gastrostomy for those procedures performed in two stages. Of note, there were only three cases who underwent an ERCP more than 1 week after the therapeutic EUS procedure. Clinical success was achieved in all cases with technical success (100%). The median length of hospitalization was 3 days (range 2–8).

**Table 1:** Technical characteristics of the endoscopic ultrasound‐directed transgastric endoscopic retrograde cholangiopancreatography (EDGE) procedure in patients with a personal background of gastric bypass

Feature	Data
Technique of LAMS insertion (*n*, %)	Wire‐guided (28, 90.3) Freehand (3, 9.7)
Location of the endoscopic anastomosis (*n*, %)	Gastro‐gastric (24, 77.4) Jejuno‐gastric (7, 22.6)
Axis of the stent (*n*, %)	Oblique (22, 71) Vertical (6, 19.4) Horizontal (3, 9.7)
Number of EDGE steps (*n*, %)	Two‐stage procedure (26, 83.9) One‐stage procedure (5, 16.1)
Number of punctures, median (range)	1 (0–2)

LAMS, lumen‐apposing metal stent.

### Anastomosis‐related difficult ERCP

There were 10 difficult ERCPs (32.3%) due to the need of performing LAMS dilation to 15–16.5 mm (*n* = 8), >5 min to achieve a short position in the duodenum (*n* = 8) and the failure to pass the endoscopic anastomosis with a duodenoscope needing a forward‐viewing endoscope (*n* = 3). These features were overlapped in most cases as all patients requiring a dilation needed more than 5 min to achieve a short position with the duodenoscope. In addition, the three patients in whom the ERCP was performed using a forward‐viewing gastroscope with a hood presented with a jejuno‐gastric anastomosis (*n* = 2) or a gastro‐gastric anastomosis with the proximal excluded stomach (*n* = 1), leading to a retroflexed position of the duodenoscope while trying to pass the LAMS. There was only one patient with a long procedure time not requiring a LAMS dilation. He was a male patient requiring 20 min to achieve a short position in the excluded duodenum due to a jejuno‐gastric anastomosis with a vertical axis in the excluded antrum leading to a narrow space to manipulate the duodenoscope and pass the pylorus.

Considering patients with a gastro‐gastric anastomosis in the proximal‐excluded stomach, the stent had a horizontal axis (*n* = 3, 75%), leading to a retroflexed position and a challenging passage through the LAMS. Regarding patients with a jejuno‐gastric approach, most of them had the anastomosis with the distal‐excluded stomach (*n* = 4, 57.1%) with a vertical LAMS axis (*n* = 5, 71.4%).

**Figure 3:**
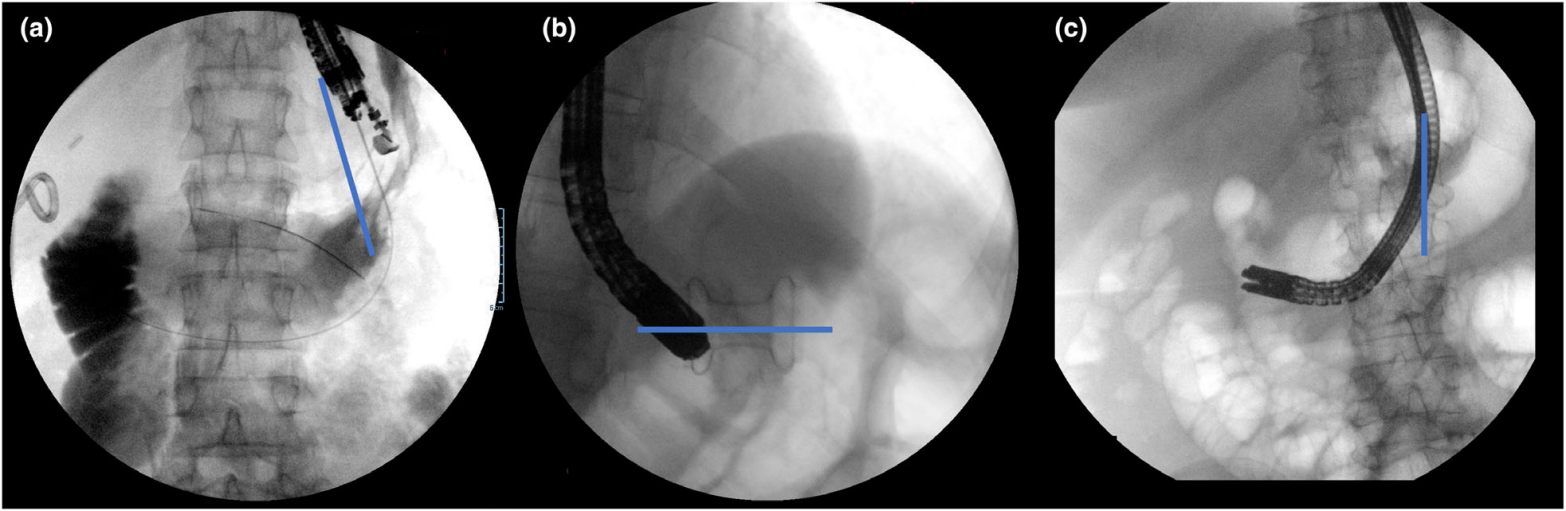
Three endoscopic ultrasound‐guided anastomosis. (a) Gastro‐gastric anastomosis in the middle‐excluded stomach with an oblique axis. (b) Tentative of passing through a gastro‐gastric anastomosis in the proximal stomach. The horizontal axis of the stent located in the subcardial region leads to a difficult manipulation of the duodenoscope. (c) A narrow space in the excluded antrum of a patient who underwent a jejuno‐gastric endoscopic anastomosis with a vertical axis leads to a difficult positioning of the scope during the intervention.

**Figure 4:**
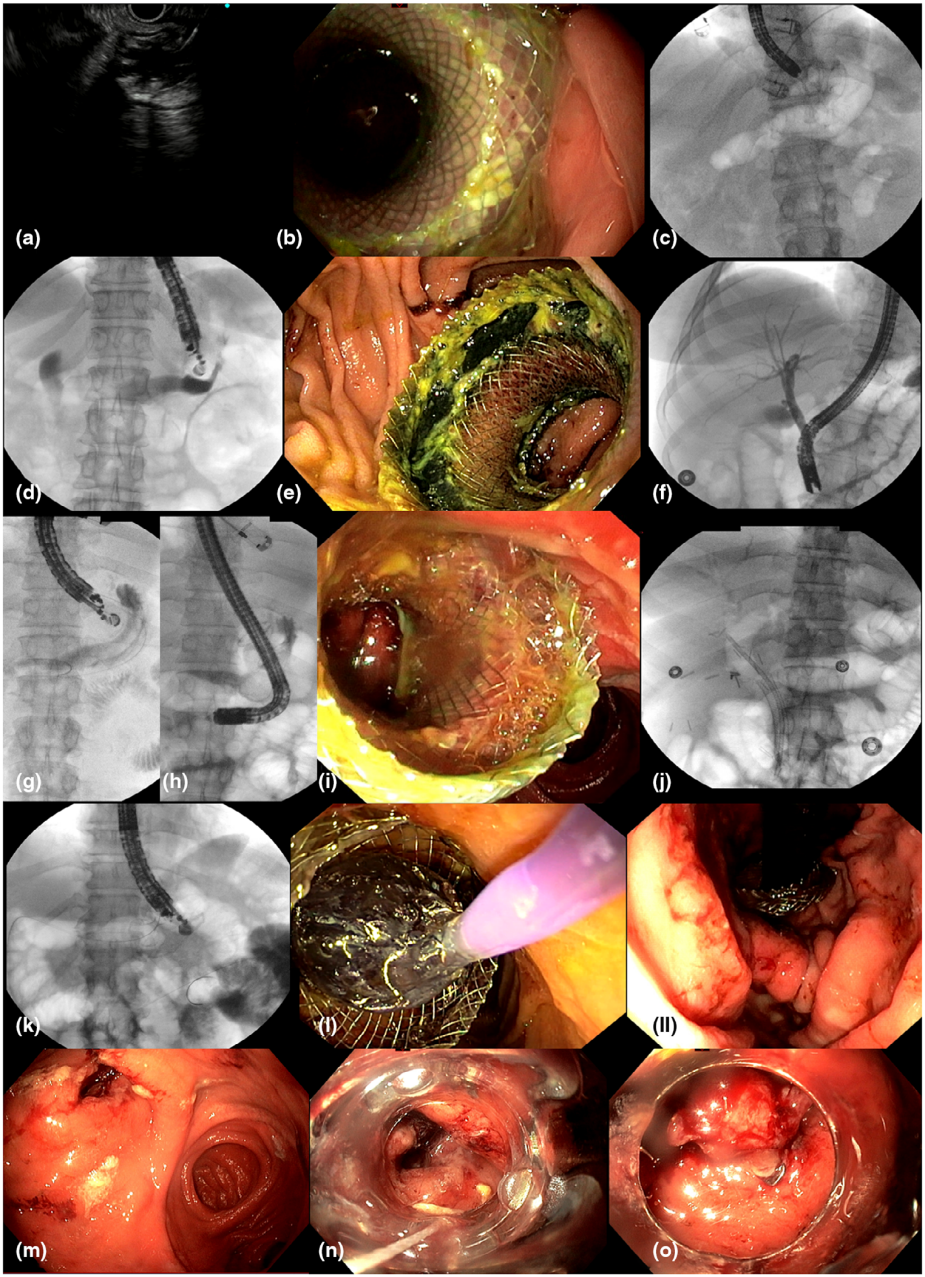
(a) Endoscopic ultrasound (EUS)‐guided puncture of the proximal excluded stomach. The lumen‐apposing metal stent (LAMS) presented a horizontal axis during endoscopic retrograde cholangiopancreatography (ERCP) under endoscopic (b) and fluoroscopy control (c). (d) EUS‐guided puncture with further opacification of the middle‐excluded stomach using a jejuno‐gastric route (e), difficult ERCP passing through a vertical LAMS (f). (g, h) Fluoroscopy images of an EUS‐guided puncture of the middle‐excluded stomach using an oblique axis with further ERCP (i) passing through an oblique LAMS (j). Difficult ERCP cases due to a LAMS placed in the distal stomach (k) requiring endoscopic dilation (l), and a challenging passage into the duodenum with a retroflexed position in the gastric cavity during ERCP (ll). (m) Endoscopic view of a jejuno‐gastric endoscopic anastomosis closed by using an over‐the‐scope clip (n, o).

At univariable analysis (Table 2), the axis was regrouped in horizontal/vertical and oblique, and the anastomotic site was regrouped in proximal/distal‐ and middle‐excluded stomach. All patients with a horizontal/vertical axis of the stent had a difficult ERCP (*P* < 0.001) and the axis was statistically associated to the anastomotic location (proximal/distal stomach; *P* < 0.001; Fig. 3), thus the axis was a confounding factor and not included in the multivariable model. At multivariable analysis adjusted by two‐stage procedures, the independent risk factors associated with a difficult ERCP were the jejuno‐gastric anastomotic location (85.7% vs. 16.7%; odds ratio [OR_a_] 31.875; 95% confidence interval [CI] 1.649–616.155; *P* = 0.022), and the anastomosis to the proximal or distal excluded stomach (70% vs. 14.3%; OR_a_ 22.667; 95% CI 1.676–306.570; *P* = 0.019; Fig. 4). The final model excluding the confounding factor achieved a Nagelkerke R‐squared index of 0.644.

**Table 2:** Univariable and multivariable analyses of risk factors for difficult anastomotic‐related endoscopic retrograde cholangiopancreatography (ERCP) in patients who underwent an endoscopic ultrasound‐directed transgastric ERCP procedure

Feature	Total cohort, *n* (%)	Risk of difficult ERCP	Univariable analysis		Adjusted multivariable analysis	
			OR (95% CI)	*P*‐value	OR_a_ (95% CI)	*P*‐value
Jejuno‐gastric anastomotic location	7 (22.6)	85.7% vs. 16.7%	30 (2.794–322.090)	0.002*	31.875 (1.649–616.155)	0.022*
Proximal/distal excluded stomach	10 (32.3)	70.0% vs. 14.3%	14 (2.262–86.662)	0.002*	22.667 (1.676–306.570)	0.019*
Horizontal/vertical axis	9 (29.0)	100% vs. 4.5%	–	–	–	–

* Statistically significant.

The axis was a confounding factor; thus, it was not included in the final model.

CI, confidence interval; OR_a_, odds ratio adjusted by two‐stage procedures.

There was only one complication (3.2%) graded AGREE IIIb in a patient with a bypass who underwent an uneventful EUS‐guided jejuno‐gastric anastomosis in the distal excluded stomach. Five days after, an ERCP was performed, and the passage of the LAMS was challenging due to the retroflexed and curved position of the endoscope despite stent dilation. An anastomotic dehiscence of the gastrojejunal surgical anastomosis occurred during the duodenoscope manipulation requiring emergency surgery with conversion to successful LA‐ERCP. The patient recovered well and was considered as an EDGE technical failure. Of note, there were no complications related to the creation of the EUS‐guided anastomosis in the whole cohort, and no other cases of LAMS dislodgement or migration.

### Follow‐up

All patients underwent a follow‐up endoscopy to remove the LAMS at a median of 31.5 days (range 6 days–14 weeks) after ERCP. The stone clearance rate in patients with biliary stones was 94.7%. Endoscopic closure of the anastomosis was performed by over‐the‐scope clip (Ovesco, Tübingen, Germany) in five cases with a jejuno‐gastric route (16.1%) and argon plasma coagulation was done in three cases (9.7%). Fistula closure was not possible in two patients with a jejuno‐gastric anastomosis. There was only one case of persistent gastro‐gastric fistula (3.2%) in a median follow‐up of 4 months (2–18), and it was successfully treated endoscopically. The remaining cases had spontaneous closure of the anastomosis. Overall, there was no weight regain (67 vs. 69 kg, *P* = 0.465). No delayed AEs were observed.

## DISCUSSION

In the present study of a homogeneous cohort of 31 patients, the jejuno‐gastric anastomotic location and the anastomosis to the proximal‐ or distal‐excluded stomach were independent risk factors for anastomosis‐related difficult ERCP. All patients with a horizontal or vertical axis of the LAMS also had a difficult ERCP (confounding factor, *P* < 0.001). Overall, the technique was effective and safe, with a very low AE rate. The rate of persistent fistula was 3.2% during a median follow‐up of 4 months even if endoscopic closure following LAMS removal was not performed in most cases.

Therapeutic EUS techniques are booming in patients with altered anatomy.^18^, ^19^ Recently, the ESGE guidelines recommend the use of saline instillation for EDGE, concluding that the “free‐hand” technique is nowadays mostly employed.^20^ However, special attention should be paid not to deploy the LAMS too caudally in the antrum or distal gastric body as these positions may lead to difficult passing. In our experience, the proximal bypassed stomach is almost always located with the scope in the subcardial region, and this is usually the most dilated area of the excluded gastric cavity. Thus, the gastro‐gastric anastomosis is probably easier in this location (but not further ERCP). Conversely, the middle‐excluded stomach is closer to the surgical gastrojejunal anastomosis and not so dilated, leading to a more challenging EUS‐guided gastro‐gastric procedure but with a probable easier manipulation of the duodenoscope during subsequent ERCP. In addition, the postsurgical anatomy may vary greatly from one patient to another, particularly the size of the gastric remnant. In our study, we performed a puncture with opacification first to understand the patient's anatomy and decide the best location of the endoscopic anastomosis. Furthermore, the middle‐excluded stomach can be more difficult to dilate using saline than the proximal or distal gastric cavity, and the region is much more fixed compared to a jejunal limb during EUS‐guided gastroenterostomy. Thus, we prefer to avoid filling with saline and perform the EUS‐guided technique that allows the perforation of the desired location even if the target cavity is not dilated.

Although the technical success of the EDGE has been high in our study (96.8%), this percentage may not be faithful to reality of this complex procedure with a wide range of difficulty levels. Indeed, the definition of “difficult ERCP” could often be related to biliary cannulation, with reported scores grading this procedure.^21^ However, there is no grading or intraprocedure definition of difficult EUS‐guided interventions using LAMS. We decided to use a composite criterion of anastomosis‐related difficult ERCP including a time criterion, however, to integrate the “number of contacts” with the LAMS in a similar way as to papilla cannulation could have been a better approach. In our practice, we do not dilate the LAMS systematically, but only in case of difficult passage of the duodenoscope; this is the reason why we have also considered this criterion in the definition.

This is the first study analyzing the risk factors of difficult EDGE to the best of our knowledge. Although there was some degree of overlap within the analyzed potential risk factors, the jejuno‐gastric anastomosis and the location of the LAMS in the proximal/distal stomach were independent features associated with a difficult ERCP in a multivariable analysis. The model was adjusted by two‐stage procedures, as the one‐stage EDGEs are considered a different setting, with a higher risk of stent‐related AEs^22^ except when stent fixation is performed.^23^ Indeed, when the scope is in the subcardial position the proximal stomach is detected by EUS and a horizontal axis of the LAMS is frequent, while the transjejunal position frequently leads to a vertical anastomosis with the excluded antrum.

The limitations of the present study besides the retrospective design and the previously discussed points related to the definition of the main outcome are the limited follow‐up time and relatively wide interval of time between the EUS‐guided procedure and the ERCP. The strengths of the research are however the use of a prospectively collected database in a short period of time and the homogeneous cohort of patients who underwent a standardized procedure.

In conclusion, special attention should be paid to creating an EUS‐guided gastro‐gastric anastomosis before transgastric ERCP procedures. The jejuno‐gastric route and the anastomosis with the proximal or distal excluded stomach should be avoided.

## CONFLICT OF INTEREST

Author E.P.‐C.‐R. holds a consultancy agreement with Boston Scientific and is an Associate Editor of *Digestive Endoscopy*. L.M. is a consultant for Taewoong Medical and received speaker's fees from Olympus Belgium and Olympus Europe. The other authors declare no conflict of interest for this article.

## FUNDING INFORMATION

None.

## Supporting information


**Video S1** Endoscopic ultrasound (EUS)‐directed transgastric endoscopic retrograde cholangiopancreatography procedure (EDGE) in a patient with a biliary leak in a one‐stage procedure. Two EUS punctures were needed to decide the better location and axis of the anastomosis.
